# Exploring the Hypocholesterolemic Potential of a *Fucus vesiculosus* Extract: Omic Insights into Molecular Mechanisms at the Intestinal Level

**DOI:** 10.3390/md22040187

**Published:** 2024-04-20

**Authors:** Rebeca André, Rita Pacheco, Hugo M. Santos, Maria Luísa Serralheiro

**Affiliations:** 1 CBIOS—Research Center for Biosciences & Health Technologies, Universidade Lusófona, Campo Grande 376, 1749-024 Lisboa, Portugal; 2 Department of Chemical Engineering, ISEL—Instituto Superior de Engenharia de Lisboa, Rua Conselheiro Emídio Navarro, 1, 1959-007 Lisboa, Portugal; 3 Centro de Química Estrutural, Institute of Molecular Sciences, Universidade de Lisboa, 1749-016 Lisboa, Portugal; 4 LAQV@REQUIMTE, Department of Chemistry, NOVA School of Science and Technology, Universidade NOVA de Lisboa, 2829-516 Caparica, Portugal; hms14862@fct.unl.pt; 5 PROTEOMASS Scientific Society, Madan Park, Rua dos Inventores, 2825-182 Caparica, Portugal; 6 BioISI—Biosystems & Integrative Sciences Institute, Faculdade de Ciências, Universidade de Lisboa, 1749-016 Lisboa, Portugal; mlserralheiro@fc.ul.pt; 7 Department of Chemistry and Biochemistry, Faculdade de Ciências, Universidade de Lisboa, Campo Grande, C8 bldg, 1749-016 Lisboa, Portugal

**Keywords:** *Fucus vesiculosus*, proteomics, metabolomics, NPC1, Caco-2 cell line

## Abstract

High blood cholesterol levels are a major risk factor for cardiovascular diseases. A purified aqueous extract of *Fucus vesiculosus*, rich in phlorotannins and peptides, has been described for its potential to inhibit cholesterol biosynthesis and intestinal absorption. In this work, the effect of this extract on intestinal cells’ metabolites and proteins was analysed to gain a deeper understanding of its mode of action on lipids’ metabolism, particularly concerning the absorption and transport of exogenous cholesterol. Caco-2 cells, differentiated into enterocytes, were exposed to the extract, and analysed by untargeted metabolomics and proteomics. The results of the metabolomic analysis showed statistically significant differences in glutathione content of cells exposed to the extract compared to control cells, along with an increased expression of fatty acid amides in exposed cells. A proteomic analysis showed an increased expression in cells exposed to the extract compared to control cells of FAB1 and NPC1, proteins known to be involved in lipid metabolism and transport. To the extent of our knowledge, this study is the first use of untargeted metabolomics and a proteomic analysis to investigate the effects of *F. vesiculosus* on differentiated Caco-2 cells, offering insights into the molecular mechanism of the extract’s compounds on intestinal cells.

## 1. Introduction

Atherosclerosis, a chronic inflammatory disease of the blood vessels [1,2], is the major cause of cardiovascular disease, the leading cause of death in Europe [3]. Hypercholesterolemia is a risk factor for atherosclerosis because elevated plasma cholesterol concentrations and cholesterol accumulation in various tissues lead to the formation of arterial plaques [3,4]. One of the main approaches to reduce the risk of atherosclerosis is to lower the blood cholesterol levels, either through increased physical activity and dietary changes, such as reducing the intake of saturated fat, or through prescribed medication [3]. Recently, there has been increased scientific interest in new therapeutic strategies using functional foods to reduce hypercholesterolemia, leading to the search for new bioactive natural products.

Different seaweeds have been characterised by their high hypocholesterolemic potential [5]. Brown seaweeds are one of the world’s most consumed seaweeds and one of the most studied seaweeds in this field [6]. Several studies with different compounds, such as phlorotannins, carotenoids, and polysaccharides extracted from brown algae species, have already reported their hypocholesterolemic effect [7,8,9]. Phlorotannins, a class of bioactive polyphenolic compounds produced by brown algae, have been characterised for their potential to prevent atherosclerosis, with several studies reporting their ability to reduce blood lipid levels and total cholesterol, particularly through their ability to decrease cholesterol synthesis and intestinal absorption [9,10,11,12]. Specifically, an aqueous extract of the brown algae *F. vesiculosus*, purified by solid-phase extraction (SPE), characterised by Liquid Chromatography High-Resolution Mass Spectrometry (LC-HRMS/MS) as rich in phlorotannins and peptides, stood out for its in vitro inhibitory effect on the synthesis and absorption of cholesterol, as well as for its ability to reduce the hepatic expression of NPC1L1, which can lead to the increased biliary excretion of cholesterol [10,11,13].

*F. vesiculosus* is one of the species of brown algae consumed in Europe and used in traditional medicine to treat or prevent various diseases such as obesity, arthritis, arthrosis, hypothyroidism, atherosclerosis, hypercholesterolemia, mineral deficiency, and anaemia, among others [14,15,16,17]. Previous studies have demonstrated the potential of this seaweed in various biological activities, namely hypercholesterolemia [10,11,18], antidiabetic activities [19], anticancer activities [20,21], prevention of atherosclerosis [10], and antiviral activities [22,23], among others. This seaweed is considered to be an excellent natural source of iodine, so its consumption is contraindicated in cases of hyperthyroidism [14].

The intestine is known to play an important role in cholesterol homeostasis in terms of absorption, with cholesterol uptake and secretion by enterocytes [24]. Previous studies have reported that the dietary incorporation of seaweed-derived polysaccharides, polyphenols, and peptides has the potential to modulate mammalian gut microbiota, leading to changes in lipid transport [25]. Therefore, the aim of this work was to study the effect on intestinal cells of a purified aqueous extract of *F. vesiculosus*, previously characterised as rich in phlorotannins and peptides, in order to better understand the mechanism of action of the extract in the different processes that regulate cholesterol homeostasis at the intestinal level. To achieve this, the intestinal barrier was simulated in vitro using a Caco-2 cell line. These cells have been widely used as a model of the intestinal barrier, as they can spontaneously differentiate after approximately 21 days into a monolayer of polarized cells with morphological and functional characteristics of small intestinal enterocytes [8]. Thus, differentiated Caco-2 cells were exposed to the extract and subsequently analysed by two different omics techniques, namely a gel-based proteomics analysis and untargeted metabolomics analysis. These two techniques are considered to be promising tools that have led to remarkable results in the investigation of molecular mechanisms associated with various diseases [26,27,28]. Given the robust results obtained through metabolomic and proteomic analyses, this study has contributed to the understanding of the extract’s mechanism of action not only in the prevention of hypercholesterolemia and atherosclerosis, but also in the management of other lipid-related conditions.

## 2. Results and Discussion

### 2.1. Effect of the Purified F. vesiculosus Aqueous Extract in Enterocyte-Like Caco-2 Cells

Before the metabolomic and proteomic studies, Caco-2 cells differentiated into enterocyte-like cells were exposed to 0.25 mg/mL of a *F. vesiculosus* aqueous extract for 24 h to assess the cytotoxic effect of the extract on the cells under study. The *F. vesiculosus* extract showed no cytotoxic effect on differentiated Caco-2 cells. When the cells were exposed to different concentrations of the extract (0.2–0.8 mg/mL), cell viability was always approximately 100%.

#### 2.1.1. Metabolomic Analysis

To study the effect of the purified *F. vesiculosus* aqueous extract on the metabolites of Caco-2 cells differentiated into enterocyte-like cells, an untargeted metabolomic analysis was performed by LC-HRMS/MS. A total of 2217 metabolites were detected in this analysis. Although a large number of metabolites were detected, we specifically identified only those with a *p*-value of less than 0.05 and available MS/MS spectra. On the basis of the criterion, twelve metabolites were putatively identified by comparison with databases (Table 1).

**Figure 1:**
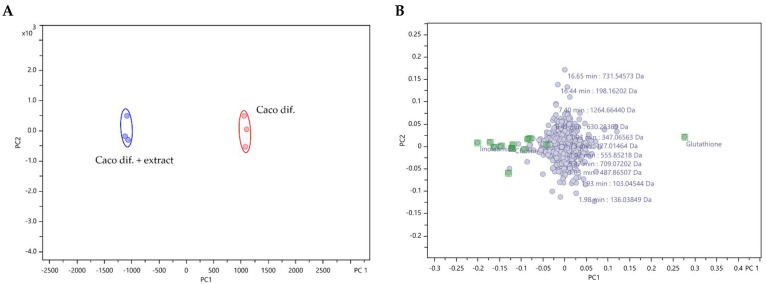
PCA analysis using Pareto scaling of LC/MS/MS untargeted metabolomic data analysis of control differentiated Caco-2 cells (Caco dif.) and cells exposed (24 h) to *F. vesiculosus* aqueous extract (0.25 mg/mL) (Caco dif + extract). (**A**) PCA score plot of PC2 versus PC1 illustrating the clustering at 98% confidence level of triplicate analysis of metabolites from control differentiated Caco-2 cells (Caco dif.—red points) and the triplicate analysis of metabolites from differentiated Caco-2 cells exposed to the extract (Caco dif. + extract—blue points). (**B**) PCA loading plot of PC2 versus PC1 of the detected compounds, with the metabolites identified in Table 1 highlighted in green.

The results of the metabolomic analysis were interpreted using an unsupervised principal component analysis (PCA). The PCA assessed the degree of metabolic differences between control cells and cells exposed to the extract, but also the similarity between replicates. Two principal components, PC1 and PC2, were extracted and in the PCA score plot (Figure 1A), a distinct separation between the control cells and extract-exposed cells was achieved at a 98% confidence level, highlighting the metabolic differences between them. Furthermore, although the PCA loading plot (Figure 1B) showed high overlap for many of the metabolites, suggesting no significant differences in the intensities of most of the detected metabolites, on the left side, eleven putatively identified metabolites (Table 1, positive FC) were recognised as the most significant in extract-exposed cells, while on the right side, glutathione (GSH) was shown to be the most prominent in control cells.

**Figure 2:**
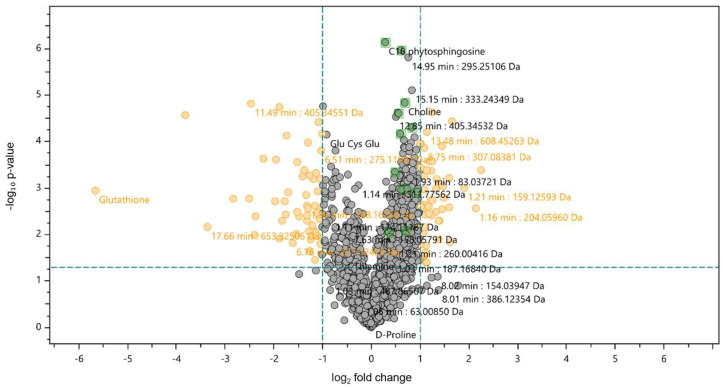
Volcano plot statistical untargeted metabolomics comparison between the control differentiated Caco-2 cells and differentiated Caco-2 cells exposed to 0.25 mg/mL of the F. vesiculosus aqueous extract, during 24 h. The volcano plot combines fold change and t-tests, where the *X*-axis represents log_2_(fold change), and the *Y*-axis represents −log_10_ (*p*-value). Grey dots indicate metabolites present at the same intensity in both cells. Right and left orange dots are up- and down-regulated metabolites in cells exposed to the extract vs. control cells, respectively.

A further statistical analysis was conducted using a t-test at a confidence level of 98% (Figure 2). Volcano plots were represented as the log_2_ fold changes in the metabolite’s abundance between the extract-exposed cells and control cells plotted against −log_10_ (*p*-value). A positive FC indicates an up-regulation of the metabolite in extract-exposed cells, whereas a negative FC signifies a down-regulation relative to the control cells.

Among the metabolites with proposed identifications, GSH emerged as the compound with a negative value of FC, shown on the left of the volcano plot, with log_2_ fold changes below −1 (Figure 2), indicating that a significant decrease in GSH occurred in cells exposed to the extract compared to control cells. However, the *F. vesiculosus* extract at 100 µg/mL showed approximately 76% antioxidant activity in a previous in vitro study, using the DPPH method [10]; the current results show that exposure of differentiated Caco-2 cells to the extract at 0.25 mg/mL induces oxidative stress and consequent glutathione depletion. This could be seen as a potential adverse effect as oxidative stress has been associated with the development of several diseases, including atherosclerosis [29], but it is worth noting that therapeutic agents causing glutathione depletion have been investigated for cancer treatment [30,31]. GSH depletion has been shown to improve the therapeutic efficacy of ROS-based therapy ferroptosis and chemotherapy by making cancer cells more susceptible to chemotherapeutic agents [30].

Among the metabolites identified in Table 1, seven were identified as fatty acid amides (2-ketoctadec-9-enamide, heptadec-2-enamide, linoleic acid hydroxamate, palmitoleoyl ethanolamide, myristamide, linoleamide, and dodecanamide). While their FC values were below 2, indicating modest changes, they exhibited a trend of increased expression in extract-exposed cells compared to control cells. These compounds are recognized for their potential health benefits and their involvement in managing various conditions, including important roles in the gastrointestinal system. Fatty acid amides have been associated with the inhibition of the enzyme ACAT, responsible for cholesterol esterification, potentially leading to the inhibition of cholesterol absorption in the intestine [11]. Moreover, the increase in fatty acid amides has also been associated with potential anti-inflammatory health benefits [32]. Previous research has suggested that the presence of this group of compounds has health benefits in the gastrointestinal tract, highlighting the benefits of an anti-inflammatory diet in obese individuals, who are at higher risk of developing cardiovascular diseases [33]. Furthermore, a similar modest trend of the increased expression of C16 phytosphingosine was also observed in cells exposed to the extract. This increase might also be related to the anti-inflammatory properties of this extract, as phytosphingosine compounds derived from natural-derived products have been reported to possess anti-inflammatory potential against chronic disorders, including cardiovascular diseases [34].

A previous untargeted metabolomic study using HepG2 cells also demonstrated that this extract led to a significant increase in the expression of several fatty acid amides [11]. As Caco-2 cells are known to be more resistant, it is possible that the changes in the expression of these lipid compounds in Caco-2 cells were less pronounced than the effects previously reported in HepG2 cells [11]. Previous results also showed that in the presence of 0.4 mg/mL of the extract, approximately 100% cell viability was obtained for differentiated Caco-2 cells, whereas only 60% cell viability was observed for HepG2 cells under the same conditions [11], highlighting that Caco-2 cells are more resistant to the effects of extract compounds.

#### 2.1.2. Proteomic Analysis

**Figure 3:**
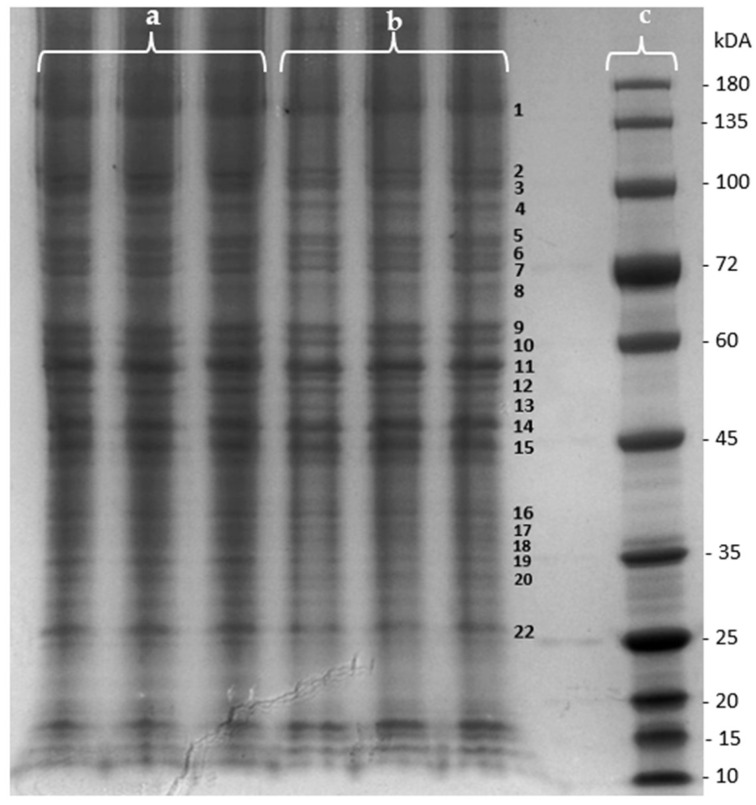
SDS-PAGE of membrane proteins extracted from differentiated Caco-2 cells under effect of (a) *F. vesiculosus* aqueous extract; (b) control; (c) protein marker.

The effect of the purified *F. vesiculosus* aqueous extract on proteins from differentiated Caco-2 cells was investigated by a gel-based proteomic analysis. Firstly, the effect of the extract on the membrane proteins of differentiated cells was evaluated using one-dimensional polyacrylamide gel electrophoresis (SDS-PAGE) for the separation and visualization of proteins prior to identification. As can be seen in Figure 3, several changes in the intensity of various protein bands were observed when proteins from control cells were compared with those from cells exposed to the extract. The determined fold changes (FCs) and *p*-values allowed us to identify differences in bands’ intensity between extract-exposed cells and the control cells (Table 2). As can be seen in Table 2, ten protein bands presented a *p*-value *<* 0.05, indicating statistically significant variations in protein intensity between the extract-exposed cells and control cells. Additionally, a positive FC signifies a higher intensity of the protein band in cells exposed to the *F. vesiculosus* extract, while a negative fold change represents a higher intensity in the control cells.

Among this set of proteins, five bands were chosen for the proteomic analysis based on their FC values. Specifically, band 19, which exhibited the highest FC value, was selected and also bands 3, 7, 9, and 10, which displayed the lowest FC values. Additionally, the zone corresponding to band 1 was also selected. In our previous study with liver cells, this extract showed the ability to decrease the mRNA and protein expression of Niemann–Pick C1-like 1 protein (NPC1L1), which has a molecular weight of approximately 145 kDa [13], corresponding to the location of band 1. NPC1L1 is the key player in dietary cholesterol uptake, transporting dietary and bile cholesterol from the intestinal lumen to the enterocyte [35]. Therefore, these six zones were excised from the SDS-PAGE of membrane proteins from both control cells and extract-exposed cells and subjected to in-gel trypsin digestion.

Peptides resulting from gel digestion were analysed in duplicate by nLC-ESI-MS/MS, followed by an Andromeda^®^ database search. In the results analysis, proteins identified in the two technical replicates were selected, and protein identification was carried out with at least two peptides and a protein FDR < 1%. The data analysis allowed the identification of 119 proteins detected only in cells exposed to the extract, 84 proteins detected only in control cells, and a total of 507 proteins present in both groups of cells.

**Figure 4:**
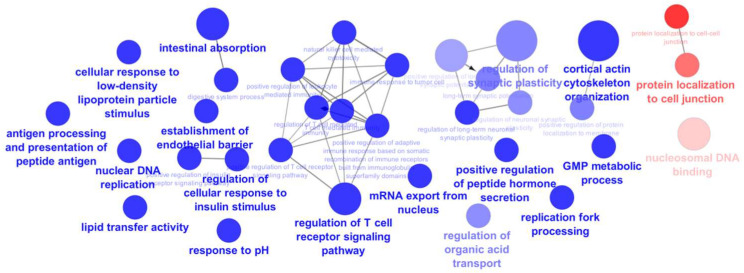
Networking of ClueGO analysis of significant enrichment GO biological process and molecular function (term *p*-value corrected with Bonferroni step down, *p*-value < 0.05), representing the enrichment terms of proteins from differentiated Caco-2 control cells (red) and proteins from differentiated Caco-2 cells exposed to *F. vesiculosus* aqueous extract (blue).

Proteins detected exclusively in cells exposed to the extract (Table S2) and those detected in the control cells (Table S1) were submitted to an analysis using ClueGo Cytoscape for the gene ontology (GO) terms related to the biological process (BO) and molecular function (MP). The network from ClueGo enrichment, shown in Figure 4, illustrates that proteins present only in cells exposed to the extract share 16 statistically significant (*p*-value < 0.05) enrichment terms.

**Figure 5:**
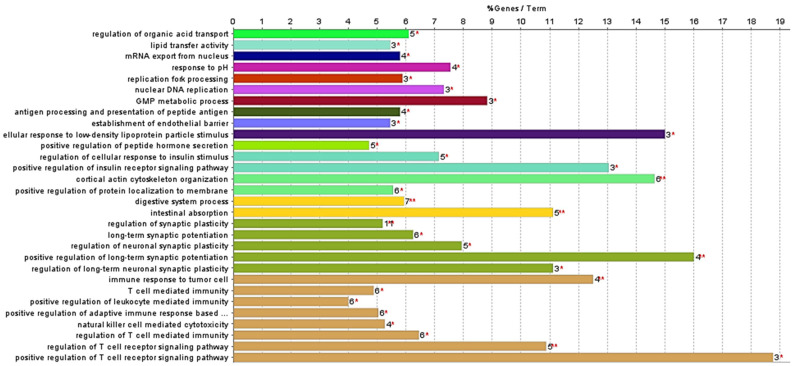
ClueGO analysis of significant enrichment GO biological process and molecular function (*p*-value term corrected with Bonferroni step down, *p*-value < 0.05) of differentiated Caco-2 cells exposed to *F. vesiculosus* aqueous extract. Bar chart representing percentage of gene by terms from differentiated Caco-2 cells exposed to *F. vesiculosus* aqueous extract. Statistical significance is calculated for both terms and groups and shown as follows: ** (*p*-value < 0.001), * (0.001 < *p*-value < 0.05).

The primary aim of this study was to highlight proteins related to the enrichment terms “intestinal absorption” and “digestive system process” (Figure 5). This focus is due to the previously reported inhibitory effect of the studied F. vesiculosus extract on the synthesis and intestinal absorption of cholesterol [10]. This is also consistent with previous studies highlighting the hypocholesterolemic potential of different brown algae [5]. However, a significant percentage of the enrichment terms annotated to the proteins from cells exposed to the extract are involved in the regulation of the T cell receptor signalling pathway (26.67%) (Figure 5). This finding might be attributable to the presence of phlorotannins in the *F. vesiculosus* extract, which have been previously associated with antitumour activity [21], but further studies are needed to explore this potential association. This was further supported by the fatty acid amides identified in extract-exposed cells, known for their anticancer and anti-inflammatory properties [36], and by the identification of the proteins Fibulin-1 (FBLN1) and sushi domain-containing protein 2 (SUSD2) exclusively in extract-exposed cells, which have been reported to be tumour suppressor proteins in colon cancer and associated with inflammation [37,38]. 

The proteins involved in intestinal absorption and the digestive system process that were identified exclusively in cells exposed to the extract were Ezrin (EZR); fatty-acid-binding protein, liver (FAB1); Niemann–Pick C1 protein (NPC1); Plastin-1 (PLS1); and solute carrier family 26 member 6 (SLC26A6). The proteins Filamin-B (FLNB) and Mucin-13 (MUC13), which are involved in the digestive system, were also identified exclusively in cells exposed to the extract. Of this group of proteins, NPC1 and FABP1 are directly related to lipids and cholesterol transport and homeostasis. The FABP1 protein is involved in lipoprotein-mediated cholesterol uptake in hepatocytes, while in the intestine, it participates in various processes related to lipid trafficking [39]. Niemann–Pick type C1 (NPC1) protein is considered a key protein in cellular cholesterol trafficking [40]; it is responsible for the transport of free cholesterol from the late endosome/lysosome to the plasma membrane and endoplasmic reticulum [41]. This protein has been related to the prevention of atherosclerosis. Although the link between NPC1 and atherosclerosis requires further study, its expression promotes the up-regulation of the ABCA1 protein, facilitating the transport of cholesterol from the late endosome/lysosome to the plasma membrane [40,41]. The ABCA1 protein is responsible for transporting cellular cholesterol to apolipoprotein A-I (apoA-I), to incorporate high-density lipoprotein cholesterol (HDL-c) particles. HDL levels are inversely associated with the risk of atherosclerotic cardiovascular disease [41,42]. Moreover, NPC1 protein is associated with atherosclerosis, as macrophages accumulate large amounts of unesterified cholesterol in the presence of advanced atherosclerotic lesions. The NPC1 protein promotes the transport of cholesterol from the late endosome/lysosome to the endoplasmic reticulum, which leads to macrophage apoptosis and plaque rupture [41]. Considering that the *F. vesiculosus* extract studied is rich in phlorotannins and peptides [10], and given that NPC1 proteins were only identified in cells exposed to the extract, we can propose that the compounds in the extract induce the expression of this protein. This effect is in line with different studies with *F. vesiculosus* seaweeds and extracts from other phlorotannin-rich seaweeds describing their hypocholesterolemic effects and their potential in the prevention of cardiovascular diseases [9,12,43,44].

## 3. Materials and Methods

### 3.1. Chemicals

All chemicals were of an analytical grade. Water, methanol (MeOH), formic acid, and acetonitrile (LC/MS grade Optima), chloroform, Pierce™ Trypsin Protease MS Grade, Pierce™ DTT (Dithiothreitol), Bolt^®^ MOPS SDS Running Buffer (20×), mini protein gel NuPAGE™ 4 to 12% Bis-Tris, and 4X Bolt™ LDS Sample Buffer were purchased from Thermo Fisher Scientific (Waltham, MA, USA). Dulbecco’s Modified Eagle’s Medium (DMEM), trypsin, glutamine, phosphate-buffered saline (PBS), and Foetal Bovine Serum (FBS) were obtained from Lonza^®^ (Verviers, Belgium). Ethanol (96%) was purchased from Carlo Erba (Peypin, France). Iodoacetamide and 3-(4,5-dimethylthiazol-2-yl)-2,5-diphenyltetrazolium bromide (MTT) were purchased from Sigma-Aldrich (Barcelona, Spain). Tris(hydroxymethyl)aminomethane and glacial acetic acid were obtained from Merck Milipore^®^ (Burlington, MA, USA, EUA). Coomassie Brilliant Blue R-250 was purchased from BIORAD^®^ (Hercules, CA, USA). A 5× SDS-PAGE Sample Loading Buffer and NZYBlue Protein Marker were purchased from Nzytech^®^ (Lumiar, Portugal).

### 3.2. Preparation and Characterization of Algae Extract

Dried *F. vesiculosus* Linnaeus seaweed harvested in the North Atlantic Ocean was purchased from Celeiro diet., Lisbon, Portugal (imported by Américo Duarte Paixão Lda, lot number 03ALG2731901). An aqueous extract of *F. vesiculosus* was prepared as described in our previous study [10]; briefly, the aqueous extract was prepared as a decoction and purified by solid-phase extraction (SPE). The characterization of the extract compounds was previously performed by liquid chromatography coupled to High-Resolution Mass Spectrometry (LC-HRMS/MS) using an Elute OLE UHPLC system interfaced with a quadrupole time-of-flight (QqToF) Impact II mass spectrometer equipped with an electrospray source (ESI) (Bruker DaltoniK GmbH, Bremen, Germany). The method description and results have been described by André et al. (2020) [10].

### 3.3. Cell Culturing and Differentiation

Caco-2 cells (ECACC 86010202), a human colorectal adenocarcinoma epithelial cell line, were cultured in DMEM supplemented with 2 mM L-glutamine and 20% FBS at 37 °C in a 5% CO_2_ atmosphere. The cultured cells were maintained at sub-confluence with trypsinization every 72 h. For cell differentiation, Caco-2 cells were seeded at a density of 2 × 10^4^ cells/cm^2^ with DMEM supplemented with 2 mM L-glutamine in a T25 flask.

### 3.4. Cytotoxicity Studies in Caco-2 Cells

The cytotoxic effect of the purified *F. vesiculosus* aqueous extract on Caco-2 cells was evaluated through the MTT viability test as described by Falé et al. (2012) [45]. The cytotoxicity study was performed in 96-well plates by exposing Caco-2 cells to different concentrations of the *F. vesiculosus* extracts in a culture medium without FBS for 24 h. The cytotoxicity mean and standard deviation were calculated using Microsoft^®^ Excel software (Microsoft^®^ Excel 2016 software, Washington, DC, USA)) from 2 × 8 replicates for each concentration.

### 3.5. Metabolomic Analysis through Liquid Chromatography Combined with High-Resolution Tandem Mass Spectrometry (LC/HRMS)

The differentiated Caco-2 cells were exposed to the purified aqueous extract of *F. vesiculosus* at 0.25 mg/mL dissolved in a culture medium without FBS (cells exposed to extract), and to a culture medium without FBS (control), for 24 h. Metabolites were extracted as described in our previous study [11]. Briefly, for each condition, 2.8 × 10^6^ cells were washed twice with cold phosphate-buffered saline (PBS). The cells were scraped and quenched with a water–methanol–chloroform solution (10:27:3) and subjected to three ultrasound cycles for 5 min. The cell suspension was then centrifuged (10 min, 10,000× *g*, 4 °C) and the supernatant containing the cell metabolites was transferred to a new tube and evaporated to dryness. The cellular metabolites from both control and extract-exposed cells were resuspended in methanol–water (1:1) and afterwards analysed by liquid chromatography coupled to high-resolution tandem mass spectrometry (LC/HRMS/MS) using an Elute OLE UHPLC system interfaced with a quadrupole time-of-flight Impact II mass spectrometer equipped with an electrospray ionization (ESI) source (Bruker Daltonics, Bremen, Germany). The analysis was carried out with an Intensity Solo 2 1.8 μm C18 100 × 2.1 nm column (Bruker Daltonics, Billerica, MA, USA) in ESI positive mode, with the following parameters: −3.5 kV and +4.0 kV; end plate offset, 500 V; nebulizer gas (N_2_), 2.0 bars; dry gas (N_2_), 8 Lmin-1; dry heater, 200 °C; collision cell energy was set to 5.0 eV. The internal calibration was performed with 250 mL of H_2_O, 50 mL of iPrOH, 750 µL of acetic acid, 250 µL of formic acid, and 0.5 mL of a 1 N NaOH solution in HPC mode. DataAnalysis 4.1 software (Bruker Daltonik GmbH, Bremen, Germany) was used to process the acquired data. The assay was performed in triplicate. The identification of the metabolites was performed taking into account the MS^2^ fragment ions, the exact mass measured, and the available reference standard mass spectral databases, namely METLIN (http://metlin.scripps.edu/, accessed on 11 September 2022), HMDB (http://www.hmdb.ca/, accessed on 15 September 2022), Bruker MetaboBASE Personal Library 2.0 (Bruker Daltonics). Statistical analysis and untargeted metabolomic analysis results were obtained using MetaboScape 4.0 software (Bruker Daltonics), as described in our previous study [11]. The MetaboScape software generates a statistical analysis, and the metabolites considered to be significantly different between the control and the cells treated with the extract were those with a significance level at a *p*-value < 0.05 and a fold change (FC) below 0.5 and above 2 in the abundance of the metabolites between the extract-exposed and the control cells.

### 3.6. Membrane Protein Extract and One-Dimension Polyacrylamide Gel Electrophoresis (SDS-PAGE)

Differentiated Caco-2 cells were exposed to 0.25 mg/mL of a purified aqueous extract of *F. vesiculosus* dissolved in a culture medium without FBS (cells exposed to extract), and to a culture medium without FBS (control), for 24 h. Cell harvesting and the extraction of the membrane protein fraction with Mem-PER Plus Membrane Protein Extraction Kit (Thermo Scientific™, Waltham, MA, USA) was performed according to the manufacturer’s instructions. The different samples of both protein fractions were separated under reducing conditions in NuPAGE 4 to 12% gradient gels (Invitrogen™, Waltham, MA, USA) using a Mini Gel Tank (Invitrogen™, Waltham, MA, USA) according to the manufacturer’s instructions. Gels were stained with 40% Coomassie R-250 blue, 50% methanol, and 10% glacial acetic acid for 1 h and destaining took place overnight with a solution of 7.5% glacial acetic acid, 10% ethanol, and 82.5% distilled water. Gels were photographed using ImageQuant LAS 50 (GE Healthcare Life Sciences^®^, Chicago, IL, USA) and the areas of the bands were determined using ImageJ software. Fold change (FC) was calculated as FC = (E/C) − 1, with the area of bands from cells exposed to the extract as E and the areas of bands from control cells as C. The areas of the bands were compared by a t-test statistical analysis using software developed by Microsoft^®^ Excel and these were considered different if the *p*-value < 0.05.

### 3.7. In-Gel Protein Digestion, Nano-LC−ESI−MS/MS, and Data Analysis

For protein identification, in-gel protein digestion was first performed as described in our previous study [46], and the resulting peptides were then analysed by an nLC-MS/MS analysis as described in a previous study [47], using an Ultimate 3000 nLC apparatus coupled to a UHR-QqTOF IMPACT HD instrument (Bruker Daltonics, Bremen, Germany) with a CaptiveSpray ion source (Bruker Daltonics, Bremen, Germany). LC-MS/MS data were processed in MaxQuant (V.1.6.10.43, Max Planck Institute of Biochemistry, Martinsried, Germany) for automated protein identification. MS raw files were analysed using MaxQuant software, version 1.6.10.43 [48], and peptide lists were searched against the human Uniprot FASTA database. A contaminant database generated by the Andromeda search engine [49] was configured with cysteine carbamidomethylation as a fixed modification and N-terminal acetylation and methionine oxidation as variable modifications. The false discovery rate (FDR) was set to 0.01 for protein and peptide levels with a minimum length of seven amino acids for peptides, and the FDR was determined by searching a reverse database. Enzyme specificity was set as the C terminal to arginine and lysine as expected using trypsin. A maximum of two missed cleavages were allowed. Data processing was performed using Perseus (version 1.6.2.3, Constellation Software, Toronto, Canada) with default settings [50].

All proteins and peptides matching the reversed database were filtered out. Subcellular localization and a gene ontology analysis were performed using STRING online resources at https://string-db.org/, accessed on 22 January 2024, and the ClueGo plug-in in Cytoscape (V3.9.0, Cytoscape Consortium, Boston, MA, USA), respectively [51].

## 4. Conclusions

The effect of a *F. vesiculosus* aqueous extract, purified by SPE, on differentiated Caco-2 cells was comprehensively characterized for the first time by untargeted metabolomic and proteomic analyses. Given the statistically significant differences, the metabolomic analysis revealed the effect of the extract in reducing glutathione and increasing fatty acid amides. Glutathione depletion was the most significant reduction in extract-exposed cells. Moreover, the extract-exposed cells exhibited increased levels of fatty acid amides and C16 phytosphingosine, compounds often associated with potential health benefits, particularly in the context of the gut where they are known to play an important role in the intricate processes of gastrointestinal lipid transport and metabolism. They are also recognized for their anti-inflammatory properties, further featuring their significance in promoting overall health and well-being. The proteomic analysis highlighted the effect of the extract in increasing the expression of several proteins, namely those having a role in lipid metabolism and transport, including NPC1 protein, one of the main proteins involved in the transport of cholesterol and directly related to the prevention of hypercholesterolemia, alongside others, which have demonstrated anti-inflammatory and antitumour properties. The current findings support the belief that a *Fucus vesiculosus* extract harbours the potential to exert beneficial effects that promote overall health and well-being. This study effectively elucidates the mechanism of action of its bioactive compounds, revealing metabolites, proteins, and pathways underlying the reduction in the risk of cardiovascular events associated with lipid disorders, while also presenting perspectives for the further exploration of its antitumour and anti-inflammatory potential.

## Figures and Tables

**Table 1:** Proposed identification of the metabolites detected by LC/HRMS/MS in ESI positive mode with increased intensity in the differentiated Caco-2 cells treated with F. vesiculosus aqueous extract compared to control cells. The tentative identification was based on exact mass, MS/MS spectra, and reference standard mass spectral databases. * Compounds without FC below 2 are not significant based on *t*-test at a confidence level of 98%.

Name	Rt	[M − H]^+^ *m*/*z*	Molecular Formula	Δ *m*/*z* (Da)	*p*-Value	FC (Extract/Control)
Heptadec-2-enamide *	17.54	268.2636	C_17_H_33_NO	0.0001	1.24 × 10^−3^	1.91
(Z)-2-ketoctadec-9-enamide *	14.57	296.2583	C_18_H_33_NO_2_	−0.00008	0.50 × 10^−4^	1.78
Linoleic acid hydroxamate *	13.11	278.2478	C_18_H_33_NO_2_	−0.00008	8.42 × 10^−3^	1.64
Choline *	1.12	104.10703	C_5_H_13_NO	−0.16	1.50 × 10^−5^	1.61
3-Hetosphingosine *	15.47	298.2739	C_18_H_35_NO_2_	−0.00010	1.05 × 10^−3^	1.59
Palmitoleoyl ethanolamide *	13.62	280.26334	C_18_H_35_NO_2_	−0.00006	0.1 × 10^−5^	1.54
Myristamide *	16.28	228.23232	C_14_H_29_NO	0.00012	2.5 × 10^−5^	1.49
Linoleamide *	14.33	280.2633	C_18_H_33_NO	−0.00001	1.73 × 10^−4^	1.47
Dodecanamide *	14.13	200.2008	C_12_H_25_NO	0.00013	4.68 × 10^−4^	1.40
Glutathione oxidized *	1.92	613.1532	C_20_H_32_N_6_O_12_S_2_	1.61	2.35 × 10^−3^	1.35
C16 phytosphingosine *	11.47	290.2691	C_16_H_35_NO_3_	−1.07	0.10 × 10^−5^	1.22
Glutathione	1.94	308.0908	C_10_H_17_N_3_O_6_S	0.0016	1.17 × 10^−3^	−50.45

**Table 2:** The *p*-values of and fold changes in the protein band intensity.

Band	Fold Change (Extract/Control)	*p*-Value
1	−0.051	0.306
2	0.157	0.369
3	−0.715	0.009
4	−0.236	3.26 × 10^−4^
5	−0.198	0.057
6	−0.044	0.390
7	−0.393	0.011
8	−0.357	0.044
9	−0.527	1.18 × 10^−4^
10	−0.415	0.001
11	0.024	0.412
12	0.146	0.231
13	−0.362	0.008
14	−0.317	0.002
15	−0.158	0.124
16	−0.065	0.349
17	−0.099	0.273
18	−0.359	0.005
19	1.382	4.97 × 10^−6^
20	0.028	0.334
21	0.040	0.232

## Data Availability

The original contributions presented in the study are included in the article/supplementary material, further inquiries can be directed to the corresponding authors.

## Supplementary Materials

The following supporting information can be downloaded at: https://www.mdpi.com/article/10.3390/md22040187/s1, Table S1: List of genes identified in control cells. Table S2: List of genes identified in cells exposed to the extract.
