# Supplementary material for: Exploring the Hypocholesterolemic Potential of a Fucus vesiculosus Extract: Omic Insights into Molecular Mechanisms at the Intestinal Level
Source: Mar Drugs. 2024 Apr 20;22(4):187. doi: 10.3390/md22040187 (PMC11050770; doi:10.3390/md22040187)
Supplement: Supplementary file 1 [file marinedrugs-22-00187-s001.zip › marinedrugs-2956677-supplementary.pdf]

Table S1- List of genes identified in control cells

Genes identified in control cells	
DBNL	PGAM1;PGAM4
DECR1	SLC7A7
VPS35	MTHFD1
LMO7	EWSR1
PGAM5	VAPB
PRKRA	RPL7
DSP	CBX3
DPYSL2	EHD1
RPS5	LSR
ATP6V1B2	RBM7
AMBP	RPL11
SLC25A6;SLC25A4	KATNAL2
BLMH	RPL8
VPS26A	PLRG1
KRT1	PNO1
API5	COLGALT1
OCLN	TMEM192
GNAS	TXN
PTPRF	TXNL1
MRPL19	SFN
HIST1H1E	HIST1H1D
LMBRD1	LMNA
PRPF19	PEG10
RPA2	DSG1
FABP5	RSL1D1
EMD	PSME2
QPRT	RPL6
HSD17B11	ENOPH1
RPL18A	HIST1H4A
XRN2	BCL2L13
TBL1XR1	NUP50
SND1	ADRM1
RBM47	NARS
IGFBP7	RPS16
SRPRB	HNRNPH3
RPL18	PSMC1
STAU1	CLN3
RPS8	PAK2
CRYGS	EEF1G
ENSG00000268083	H1F0
ACAD11	S100A9
TOP1	PARP1

Table S2- List of genes identified in cells exposed to the extract

Genes identified in control cells		
DLG1	EZR	SNX2
PPP2CA;PPP2CB	AGT	IGHA2
PGM2	ERAP1	ADD1
QARS	CD81	COL4A3BP
SIGIRR	MCM2	IPO5
ESYT1	DBN1	HMGCS1
ATP6V0D1	MCM4	GLOD4
SLC23A1	OSBP	ATP6V1D
NUCKS1	HTATSF1	GBA
BDH1	EPB41L1	CEACAM1
CTNNBL1	SLC29A1	CTNNA2
YWHAG	AHNAK	EPS15L1
CD276	DKFZp566H1924;NPTN	GALNS
KDELC2	TRMT6	ITGA2
SUSD2	ESYT2	MFI2
NSF	CAPN5	NRD1
GNAI2	ALYREF	CASK
LGMN	RDH14	CPPED1
SNX27	LUC7L3	NPEPPS;NPEPPSL1
KPNA2	TMEM87A	FBXO2
SLC35F6	ASPH	TRIP10
SMARCD2	STS	NPC1
NUP93	RDX	ADA
SLC7A5	FBLN1	MAL2
NAE1	MYOF	STX4
FABP1	HAAO	PVRL2
HNRNPF	PLS1	APP
GALNT6	FKBP10	ANXA11
TMX3	CALR	FAM49B
NUP160	PCNA	HLA-A
ERBB3	PDPR	PVR
CEACAM5	PRKAR1A	SLC26A6
COQ9	LCP1	SUCLG1
SLC38A3	DPP7	OAT
ALDH16A1	MPZL1	HIST1H2BN
CLU	SERINC1	
CD63	HDAC1	
IPO7	NUP133	
SNRPA	MUC13	
APLP2	ITGA6	
HMGCL	DHTKD1	
TMEM87B	FLNB	
